# P-1677. Nanoparticle Capture of Urinary Lipoarabinomannan for Diagnosing Childhood Tuberculosis

**DOI:** 10.1093/ofid/ofaf695.1851

**Published:** 2026-01-11

**Authors:** Brendan Mullen, Najeeha Iqbal, Kumail Ahmed, Tania Thomas

**Affiliations:** University of Washington, Seattle, Washington; Aga Khan University, Karachi, Sindh, Pakistan; Aga Khan University, Karachi, Sindh, Pakistan; University of Virginia, Charlottesville, Virginia

## Abstract

**Background:**

Non-respiratory biomarkers for tuberculosis (TB), such as the urinary lipoarabinomannan (LAM) antigen, are crucial for diagnosing TB in at-risk pediatric populations. Currently available urine assays to detect LAM as a diagnostic test for TB have limited sensitivity in pediatric populations. We aimed to evaluate the Ceres TB-Nanotrap as a urine concentration step to augment detection of LAM antigens in urine.

**Methods:**

Urine specimens were obtained from children 1-18 years of age with presumptive TB (cases) and non-TB controls from outpatient clinics in Karachi, Pakistan. An Alere lateral flow (LF)-LAM test was performed before and after concentrating urine with magnetic TB-Nanotrap particles. Band intensity was measured via visual grading and digital quantification (using Image-J software) and performance characteristics were calculated for unconcentrated and concentrated urine.

**Results:**

Urine LAM sensitivity by visual grading was 4.5% (95% Confidence Interval [CI]: 0.3 – 18.5) pre-concentration and 50.0% (95% CI 30.0 – 70.0) post-concentration. Sensitivity by digital quantification was 0.0% (95% CI 0.0 – 15.4) pre-concentration and 63.9% (95% CI 42.8 – 81.4) post-concentration. Specificity was high with both methods (visual grading 90.0% [62.8 – 99.4] pre-concentration, 90.0% [62.8 – 99.4] post-concentration; digital analysis 80.0% [44.4 – 97.5], 90.0% [62.8 – 99.4] respectively). For specimens from TB cases, digital analysis showed an increase in median LF-LAM band intensity from 0.0 arbitrary units (AU) (IQR 0.00 – 32.5) in unconcentrated samples to 98.4 AU (IQR 34.9 – 212.2) in concentrated samples.

**Conclusion:**

Concentration with TB-Nanotrap greatly increased sensitivity of urine LAM detection without change in specificity. Digital quantitative analysis further increased sensitivity. Incorporating this concentration step into urine LAM may improve diagnostic accuracy of the test. Use of digital quantitative analysis of urine LAM may increase sensitivity and allow for more precise quantification of urine LAM. Future work should explore the use of TB-Nanotrap concentration in other populations that are challenging to diagnose.

**Disclosures:**

All Authors: No reported disclosures

